# Mutation Signatures and In Silico Docking of Novel SARS-CoV-2 Variants of Concern

**DOI:** 10.3390/microorganisms9050926

**Published:** 2021-04-26

**Authors:** Nariman Shahhosseini, George (Giorgi) Babuadze, Gary Wong, Gary P. Kobinger

**Affiliations:** 1 Département de Microbiologie-Infectiologie et d’Immunologie, Université Laval, Québec City, QC G1V4G2, Canada; gary.wong@crchudequebec.ulaval.ca (G.W.); gary.kobinger@crchudequebec.ulaval.ca (G.P.K.); 2 Department of Biological Sciences, Sunnybrook Research Institute, University of Toronto, Toronto, ON M4N3M5, Canada; george.babuadze@utoronto.ca; 3 Institut Pasteur of Shanghai, Chinese Academy of Sciences, Shanghai 200031, China; 4 Department of Medical Microbiology, University of Manitoba, Winnipeg, MB R3E0J9, Canada; 5 Department of Immunology, University of Manitoba, Winnipeg, MB R3E0T5, Canada; 6 Department of Pathology and Laboratory Medicine, School of Medicine, University of Pennsylvania, Philadelphia, PA 19104-4238, USA

**Keywords:** SARS-CoV-2, variants of concern, mutation, molecular interaction, binding free energy

## Abstract

One year since the first severe acute respiratory syndrome coronavirus 2 (SARS-CoV-2) was reported in China, several variants of concern (VOC) have appeared around the world, with some variants seeming to pose a greater thread to public health due to enhanced transmissibility or infectivity. This study provides a framework for molecular characterization of novel VOC and investigates the effect of mutations on the binding affinity of the receptor-binding domain (RBD) to human angiotensin-converting enzyme 2 (hACE2) using in silico approach. Notable nonsynonymous mutations in RBD of VOC include the E484K and K417N/T that can be seen in South African and Brazilian variants, and N501Y and D614G that can be seen in all VOC. Phylogenetic analyses demonstrated that although the UK-VOC and the BR-VOC fell in the clade GR, they have different mutation signatures, implying an independent evolutionary pathway. The same is true about SA-VOC and COH-VOC felling in clade GH, but different mutation signatures. Combining molecular interaction modeling and the free energy of binding (FEB) calculations for VOC, it can be assumed that the mutation N501Y has the highest binding affinity in RBD for all VOC, followed by E484K (only for BR-VOC), which favors the formation of a stable complex. However, mutations at the residue K417N/T are shown to reduce the binding affinity. Once vaccination has started, there will be selective pressure that would be in favor of the emergence of novel variants capable of escaping the immune system. Therefore, genomic surveillance should be enhanced to find and monitor new emerging SARS-CoV-2 variants before they become a public health concern.

## 1. Introduction

Novel viruses have been emerging sporadically. Emerging viruses are mainly of zoonotic origin and are often the result of a cross-species transmission. This can happen through several genetic variation mechanisms including mutation, recombination, and genome segment reassortment or combinations of these molecular events that cause new features and enable the virus to bind and enter into a new host cell with greater efficiency, avoid the immune system, and modify its virulence. Viruses acquire mutations over time, which can lead to the emergence of new variants. In general, RNA viruses have higher mutation rate than DNA viruses, single-stranded viruses tend to mutate faster than double-stranded viruses, and viruses with smaller genome size tend to mutate faster [1].

In the mid of December 2019, a novel coronavirus (CoV) was reported in Wuhan, China. SARS-CoV-2 is causative agent of Coronavirus disease 2019 (COVID-19) [2]. As of 11 March 2021, COVID-19 disease has affected more than 118 million people in 218 countries/territories with 2.62 million of fatal outcomes. SARS-CoV-2 is a single, positive stranded RNA virus with genome size ranging between 29.8 and 29.9 kb, which codes for ORF1a, ORF1b, Spike (S), ORF3a, ORF3b, Envelope, Membrane, ORF6, ORF7a, ORF7b, ORF8, ORF9b, ORF14, Nucleocapsid, and ORF10 proteins. In S-protein structure, RBD is responsible to dock the virus to its receptors on human cells called hACE2 [3].

Three nomenclature systems for SARS-CoV-2 have been proposed including; (i) Nextstrain system has identified 11 major clades as 19A, 19B, and 20A-20I, (ii) Rambaut et al. system (known as PANGOLIN lineage) has identified five major lineages as A, B, A.1, B.1.1, and B.1.177 [4], and (iii) The Global Initiative on Sharing All Influenza Data (GISAID) has classified all SARS-CoV-2 sequences into currently seven major clades represented by their mutation signatures. Based on GISAID classification, the clade L is considered as wild-type (WT) variant, which consists of initial isolates from China (including reference genome) or other countries mainly involved in the first stage of pandemic; the clade S is defined by L84S mutation in NS8; the clade V is defined by G251V mutation in NS3; the clade G is defined by D614G mutation in S-protein (dominant isolate since spring 2020) [5]. Subsequently, three clades ‘GH, GR, GV’ descended from the clade G, while each one has identical mutations in addition to D614G. Therefore, the clade GH is defined by Q57H mutation in NS3, the clade GR is defined by G204R mutation in N-protein; and the clade GV is defined by A222V mutation in S-protein. Based on GISAID nomenclature system, the eighth clade is named clade ‘O’, however, it is not well defined as a clade, and is merged into clade V in some GISAID sources [6].

CoVs have proofreading function during replication, which results in fewer mutations and higher accuracy in virus replication than most RNA viruses [7]. Therefore, CoV replication accuracy is mainly determined by the 3′-to-5′ exoribonuclease encoded in nonstructural protein 14 (nsp14-ExoN) that proofreads RNA during replication through excision of mismatched incorporated nucleotides [8]. Nevertheless, the rapid global spread of SARS-CoV-2 means high levels of viral replication that consequently increases the chance of mutation events. As a result, several mutations have been detected in SARS-CoV-2, leading to the emergence of novel variants. Therefore, the first SARS-CoV-2 VOC was detected in the UK (UK-VOC) in October 2020, and very soon became the dominant circulating viral variant in numerous countries around the world (90 countries/territories as of the 10 February 2021). The UK-VOC is known as ‘VOC-202012-01, and also as 20I/501Y.V1 or lineage B.1.1.7 [9]. Second, another SARS-CoV-2 variant was first identified in a patient from South Africa (SA) in October 2020 known as 20H/501Y.V2, lineage B.1.351, or simply called the SA-VOC. As of 10 February 2021, cases of SA-VOC have been reported in 40 countries [10]. Third, a novel SARS-CoV-2 variant was detected in a patient from Columbus-Ohio (COH) in December 2020, named COH-VOC, and has currently become the major circulating variant in Columbus. The COH-VOC is known as COH.20G/501Y or lineage B.1.2 [11]. Lastly, at the beginning of 2021, a new variant of SARS-CoV-2 was detected in four travelers returning from Brazil (BR) to Japan known as 20J/501Y.V3 or lineage P.1 (B.1.1.248), or simply called the BR-VOC. As of 10 February 2021, cases of BR-VOC have been reported in 11 countries/territories [12].

In a binding process, the free energy difference between a ligand and protein is a determinant of binding affinity in silico using molecular simulations. In other words, the enormity of binding affinity is a measure for the strength of ligand-protein interaction. The FEB indicates the contribution of the residues of a protein in complex formation with a ligand. Hence, FEB calculation can predict the protein-ligand binding affinities. In terms of binding affinity between a virus protein and host cell receptors, how rigorous virus residues are able to dock to the receptors on the surface of host cell can be applied to determine the virus ability to increase contagiousness [13,14]. Furthermore, it is known that the length of the hydrogen bond (H-bond) between viral and host amino acids can influence the likelihood of molecular interactions, which is required for the establishment of a stable complex and virus attachment to the host cells [15]. Accordingly, the cut-offs for a strong, moderate, and weak H-bond are the bond length of 2.2–2.5 Angstrom (Å), 2.5–3.2 Å, and 3.2–4.0 Å respectively [16]. Once a H-bond between a positive charge amino acid (e.g., Arg or Lys) and a negative charge amino acid (e.g., Glu or Asp) is close enough (2.5 Å), a salt-bridge bond occurs [17].

Since early 2020, the global COVID-19 vaccine R&D pipeline included nearly 157 vaccine candidates around the world [18]. The S-protein is the basis of most of these candidate vaccines. Therefore, it is of paramount importance to know whether mutations in novel SARS-CoV-2 variants can change infectivity or render vaccines and therapeutic approaches less effective. Collectively, it is crucial to surveil the emergence of novel SARS-CoV-2 variants, characterize their genome, and assess the FEB following mutations as an indicator for infectivity of different variants. Thus, the main aim of this study is to provide a framework for molecular characterization of novel variants of SARS-CoV-2 and investigate the effect of mutations on molecular interactions and the binding affinity of the RBD to hACE2 using in silico approach to shed light on their role in viral infection.

## 2. Material and Methods

### 2.1. Dataset Collection and Mutation Comparison

A total number of 70 full-length sequences of SARS-CoV-2 covering 43 countries of origin, and from different pandemic phases (phase 0; December 2019, phase 1; winter 2020, phase 2; spring 2020, phase 3; summer 2020, phase 4; fall 2020, phase 5; winter 2021), covering all clades were retrieved from the GISAID database (https://www.gisaid.org/, accessed on 1 April 2021), however, similar sequences from the same country, similar isolation phase and clade were omitted from final analysis. Additionally, the first SARS-CoV-2 sequence from Wuhan was retrieved from the GenBank (https://www.ncbi.nlm.gov, accessed on 1 April 2021) as a WT variant (reference sequence: NC-045512). For mutation analysis, multiple amino acid alignments were carried out using the ClustalW algorithm by Geneious version 11.1.2 software, and mutation sites were determined for each protein (Biomatters Ltd., Auckland, New Zealand).

### 2.2. Phylogenetic Tree Construction

The ClustalW algorithm implemented in the Geneious software version 11.1.2 (Biomatters Ltd., Auckland, New Zealand) was used to align SARS-CoV-2 sequences. To build the phylogenetic tree for the recent VOC, the Kimura 2-parameters genetic distances model and Maximum Likelihood (ML) method were selected with sorted topologies. A bootstrap value of 1000 replicates was applied to yield a robust phylogenetic tree. Analyses of the sequences were conducted using the Geneious software version 11.1.2 [19].

### 2.3. In Silico Modeling of Molecular Interaction in RBD-hACE2 Complex

The 3D homology model was constructed based on X-ray structure of SARS-CoV-2 RBD-hACE2 docked complex (PDB ID: 6vw1.1) using Oligomeric modeling implemented in the SWISS-MODEL workspace (http://swissmodel.expasy.org, accessed on 1 April 2021). A global model quality was obtained using the QMEAN scoring functions based on physicochemical properties and the secondary structure was extracted from the 3D structure using DSSP program based on H-bond estimation algorithm [20,21]. In order to evaluate the reliability of predicted 3D structures, additionally, we used RaptorX and HDock servers to reconstruct and compare the molecular structures. Then, the docked structures with the lowest root mean square deviation (RMSD) and highest docking score were selected for further investigation [22,23]. Therefore, the created PDB files were used to infer protein-protein interaction (PPI) in the docked structures of hACE2 and WT or VOC RBD using interacting residue plots generated by the DIMPLOT tool implemented in Ligplot+ v.2.2.4 (EMBL-EBI, Cambridge, UK) [24]. In order to generate Ligplot+ diagrams, Runtime parameters related to the HBPLUS program were adjusted to 4 Å to compute potential H-bonds and non-bonded contacts. However, only interactions with the length of 2.5 Å or less were considered as a strong H-bond [16].

### 2.4. Molecular Architecture and In Silico FEB of RBD-hACE2

The created PDB files were used to evaluate the effect of mutations on the binding affinity of the RBD to hACE2. For this end, Molecular Mechanics with Generalized Born and Surface Area (MM/GBSA) method implemented in the HawkDock web server (http://cadd.zju.edu.cn/hawkdock/, accessed on 1 April 2021) was employed to calculate the FEB between mutant RBD of SARS-CoV-2 variants and hACE2 in protein-ligand docking complexes [25]. The structures of PPI for each variant were predicted by combining the ATTRACT docking algorithm and the HawkRank scoring function implemented in the HawkDock, and the key residues for PPIs were highlighted by the MM/GBSA free energy decomposition [26].

## 3. Results

### 3.1. Nonsynonymous Mutations of Novel SARS-CoV-2 Variants

The UK-VOC has 3 deletions at positions H69-V70 and Y144 in S-protein. In addition, the UK-VOC contains several nonsynonymous mutations that cause seven aa substitutions at positions N501Y, A570D, D614G, P681H, T716I, S982A, D1118H in S-protein, in which N501Y mutation occurs in the key residue of RBD. Moreover, the UK-VOC contains several aa substitutions in other genomic regions, including NSP3: T183I, A890D, I1412T, NSP12: P323L, NS8: R52I, Y73C, and N-protein; D3L, R203K, G204R, S235F. In NSP6, three aa ‘SGF” are deleted between positions 106-108 (Table 1).

The SA-VOC has seven aa substitutions in the S-protein, including D80A, D215G, K417N, E484K, N501Y, D614G, and A701V, and three aa ‘LLA’ are deleted between positions 241–243. Unlike the UK-VOC, the SA-VOC does not contain the deletion at H69-V70 in the S-protein. In addition, the SA-VOC has several aa substitutions in NSP2 (R4C), NSP3 (K837N), NSP12 (P323L), NSP13 (E168D), NSP14 (S28C), and NSP15 (P205L), NS3 (Q57H, S171L), E (P71L), N-protein (T205I) (Table 1).

The COH-VOC has two mutations at positions N501Y and D614G in the S-protein, while harboring several mutations in the NSP2 (T85I), NSP3 (T181I, A256V), NSP5 (L89F, P108S), NSP6 (G258E), NSP12 (P323L), NSP14 (N129D), and NSP16 (R216C), NS3 (Q57H, G172V), NS7a (T120I), NS8 (S24L), and N-protein (P67S, P199L) (Table 1).

The BR-VOC is highly mutated in the S-gene resulting in 12 aa substitutions in the S-protein including L18F, T20N, P26S, D138Y, R190S, K417T, E484K, N501Y, D614G, H655Y, T1027I, and V1176F. In addition, several aa substitutions can be seen in the NSP3 (S370L, K977Q), NSP12 (P323L), and NSP13 (E341D), NS3 (S253P), NS8 (E92K), N-protein (P80R, R203K, G204R). In NSP6, three aa ‘SGF” are deleted between positions 106-108 (Table 1).

### 3.2. Phylogeny of Novel SARS-CoV-2 Variants

**Figure 1:**
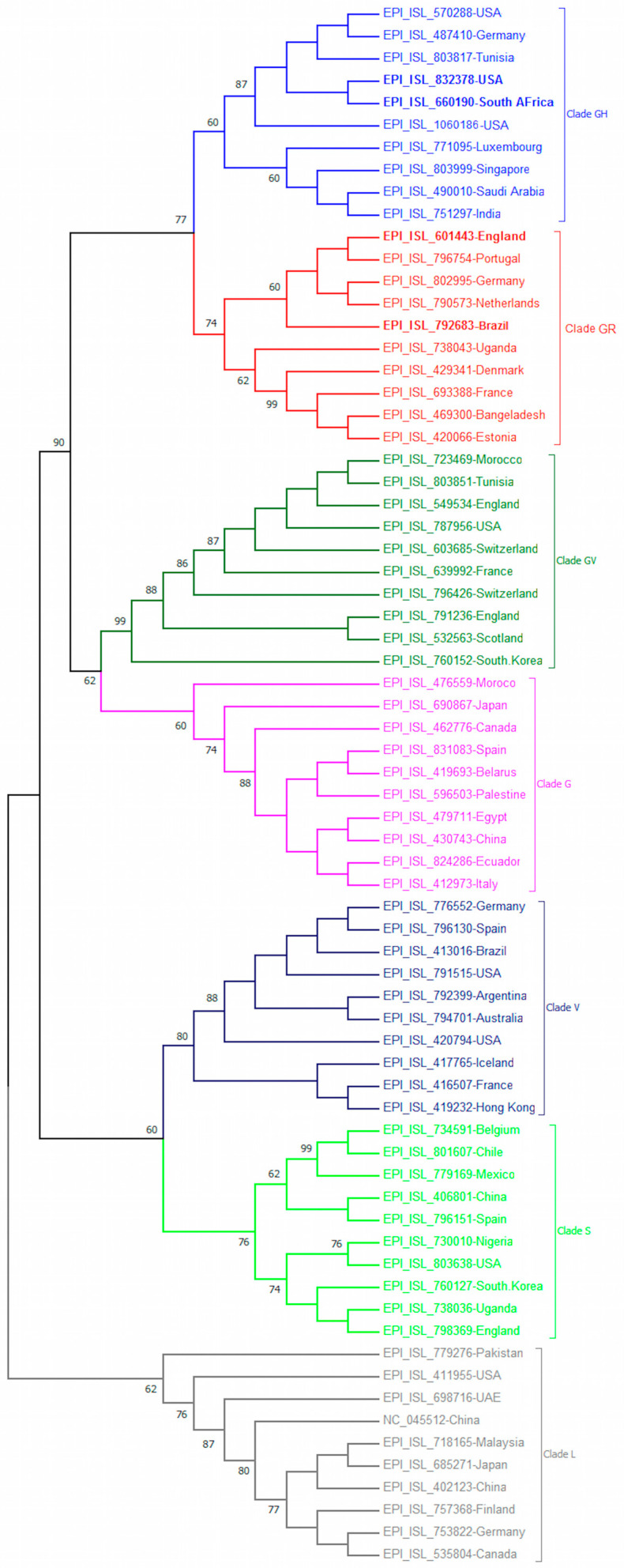
The phylogenetic tree was constructed using Geneious software. The percentage of trees in which the associated taxa clustered together is shown next to the branches.

Two emerging the UK-VOC and the BR-VOC fell in the clade GR, where the UK-VOC clustered with other isolates from Germany, Netherlands, Portugal, but formed a distant cluster from the BR-VOC. The SA-VOC and COH-VOC fell in clade GH and formed two new single phylogenetic clusters (Figure 1).

### 3.3. Molecular Interactions Between hACE2 and WT or VOC RBD

**Figure 2:**
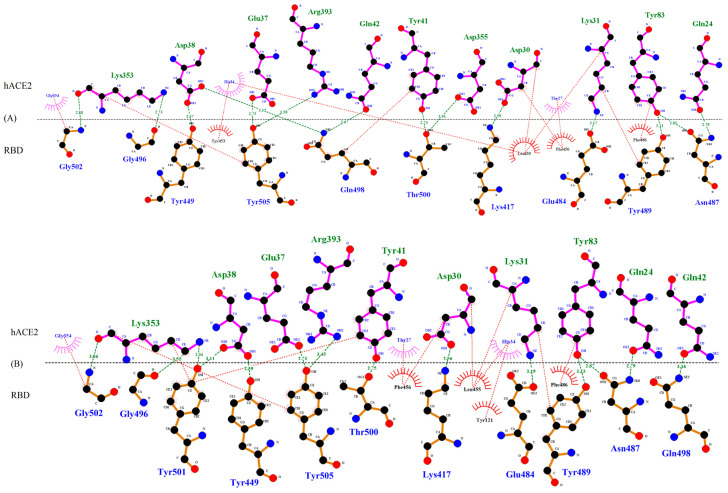

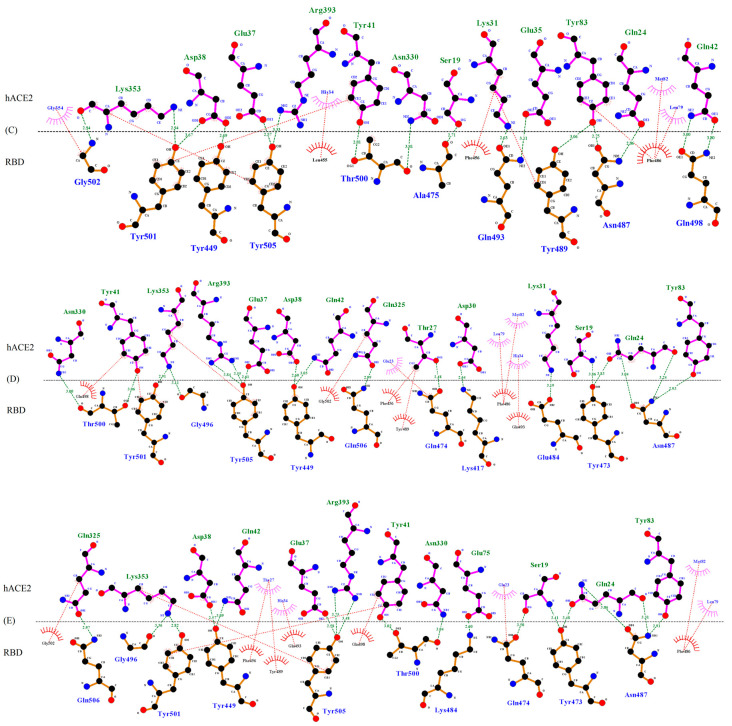
Interacting amino acids between (**A**) WT RBD-hACE-2; (**B**) UK-VOC RBD-hACE-2; (**C**) SA-VOC-RBD-hACE-2; (**D**) COH-VOC-RBD-hACE-2; (**E**) BR-VOC-RBD-hACE-2. The models were analyzed for interactions using DIMPLOT tool implemented in Ligplot+ v.2.2.4. The H-bond/salt-bridge and hydrophobic interactions are shown with green and red dots, respectively.

Homology modeling using SWISS-MODEL server determined the identity percentage between the sequences of the query and of the template as 97.09%, 96.96%, 96.84%, 96.96%, and 96.84% for WT, UK-VOC, SA-VOC, COH-VOC, and BR-VOC respectively. The molecular simulation showed that SARS-CoV-2 anchors to hACE2 (aa 19-614) of host cells by RBD that contains 193 aa (between residues 333 and 525), in which 51 aa (between residues 455 and 505) are in contact residues of RBD, known as receptor binding motif (RBM). Thus, we focus on the effect of mutations on the binding affinity of RBD to the hACE-2. Several mutations can be seen in the RBD of VOC, including K417N/T, E484K and N501Y, while two later mutations are located in contact residue responsible to directly bind to hACE2 (Figure 2).

The H-bonds and hydrophobic interactions between hACE2 and WT or VOC RBD are shown in Figure 2. In the WT, UK-VOC and COH-VOC, the residue K417 (Lys417) makes a H-bond interaction with residue D30 (Asp30) on hACE2, which is a strong bond with the length of 2.56-2.64 Å. Furthermore, the residue K417 is close enough to D30 to form a strong salt-bridge bond, which is a combination of H-bond and ionic bond. However, N417 (Asn417) in SA-VOC and T417 (Thr417) in BR-VOC do not appear to interact with hACE2, as the H-bond length is longer than 4 Å.

The residue E484 (Glu484) in the flexible loop region of RBD in WT, UK-VOC, and COH-VOC forms a H-bond interaction with residue K31 (Lys31) (H-bond lengths ranging from 3.17 to 3.19 Å). The mutation E484K in SA-VOC and BR-VOC shows different molecular interactions with hACE2. Accordingly, residue K484 (Lys484) in SA-VOC has no interaction with hACE2, while in BR-VOC, this residue forms a strong H-bond interaction (H-bond length of 2.60 Å) with residue E75 (Glu75) on hACE2, which is close enough to form a salt-bridge.

The residue N501 (Asn501) in WT forms a 4.28 Å H-bond with residue K353 (Lys353) on hACE2 (H-bonds of 4 Å or longer length are not shown in Figure 2). However, the mutation N501Y increases molecular interactions in all VOC, while the residue Y501 (Tyr501) forms a H-bond with K353 (Lys353) on hACE2 (H-bond lengths ranging from 2.82 to 2.94 Å). Further, the residue Y501 forms a H-bond with residue D38 (Asp38) in UK-VOC (bond length of 3.11 Å) and SA-VOC (bond length of 3.67 Å), while this residue in all VOC forms a stacking interaction with Y41 (Tyr41).

### 3.4. FEB of RBD-hACE2 Docked Complexes

**Figure 3:**
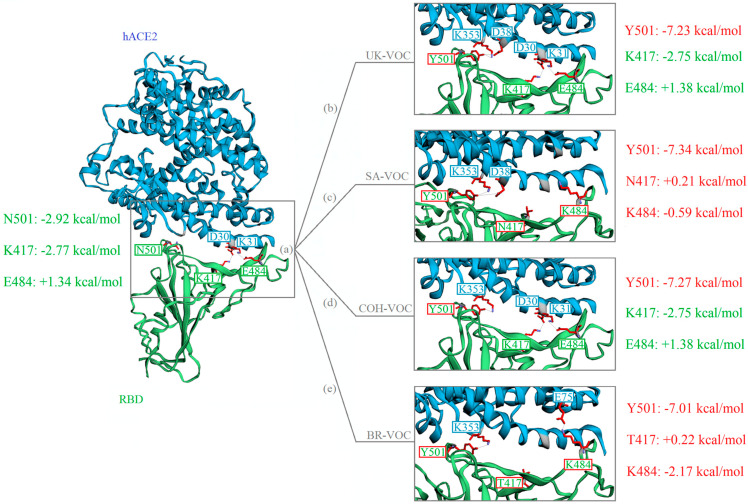
Structure of the SARS-CoV-2 RBD (green) docked with hACE2 (blue). Mutations are shown with red boxes. The contribution of residues to the total FEB (kcal/mol) in the RBD-hACE2 docked complexes for WT (**a**), UK-VOC (**b**), SA-VOC (**c**), COH-VOC (**d**), and BR-VOC (**e**) are computed using MM/GBSA method.

In silico analysis using MM/GBSA method showed that the residue K417 contributes to the total binding energy by −2.77 kcal/mol in WT variant, whereas the N/T417 mutations in VOC do not show any significant contribution to the total binding energy (+0.21 kcal/mol for SA-VOC and +0.22 kcal/mol for BR-VOC) (Figure 3).

Residue E484 in flexible loop region of RBD in WT variant contributes to binding by +1.34 kcal/mol, an unfavorable contribution to the total binding energy. However, E484K mutation decreases the FEB to −0.59 kcal/mol for SA-VOC. Interestingly, E484K mutation in BR-VOC shows a favorable contribution to the total binding energy by −2.17 kcal/mol. Therefore, considering FEB, it seems that the E484K mutation is in favor of complex formation between RBD and residue E75 on hACE2 (Figure 3).

The N501Y mutation is common in all VOC. In silico analysis demonstrated that the contribution of N501 to the total FEB in WT variant is −2.92 kcal/mol, while the Y501 mutation in this residue decreases the FEB to −7.18 ± 0.17 kcal/mol in VOC, which is in favor of complex formation by making H-bonds between RBD and residue K353 or D38 on hACE2 (Figure 3).

## 4. Discussion

Ongoing genomic characterization of the sequences will enable scientists to identify novel viral variants for further characterization. Viruses naturally mutate during replication, but the discovery of accumulating numbers of novel SARS-CoV-2 variants in the late 2020 and beginning 2021 could be due to a dramatic surge in the number of SARS-CoV-2 infected individuals during this stage of pandemic, as population size is a key determinant that affect viral diversity. As SARS-CoV-2 spreads over a larger number of humans, the more opportunity the virus has for replication and mutation, and therefore, a higher chance for the emergence of novel variants [10].

While recombination events facilitated the cross-species transmission of SARS-CoV-2 [27], mutations have demonstrated to play a critical role in the continuing evolution and emergence of novel SARS-CoV-2 variants in different phases of COVID-19 pandemic. Here, we studied the molecular evolution and mutation signatures deriving the emergence of novel SARS-CoV-2 variants in a carefully curated dataset with spatial and temporal diversity. The phylogenetic study of novel emerging VOC is important to understand the pathway of SARS-CoV-2 evolution. After a 1-year evolution period, the clustering of SARS-CoV-2 sequences revealed the spread of clades to diverse geographical regions (Figure 1), which is in contrast with distinct geographical clustering of MERS-CoV [28]. Since spring 2020, the SARS-CoV-2 clade G and then all its descendent clades ‘GH, GR, GV’ with D614G mutation have become dominant circulating variants worldwide [29]. Although it is demonstrated that the D614G does not impact on S-protein topology, variants with D614G mutation have a weaker interaction between S1 and S2 subunits of S-protein compared with WT variant, therefore, increase cleavage rate of S1–S2 subunits which facilitate virus entry into host cells [3].

Since the COVID-19 pandemic began in early 2020, a couple of variants have emerged around the globe. Emerging variants without mutations associated with enhanced transmissibility or virulence were not recognized as concerning variants. Furthermore, some variants such as mink SARS-CoV-2 variant with the Y453F mutation in RBD of S-protein known to escape neutralizing antibodies [30], however, with restricted geographical spread during outbreak (mainly detected in Denmark and the Netherlands), were also not considered as a variant of concern [31]. Conversely, some variants (e.g., UK-VOC, SA-VOC, COH-VOC, and BR-VOC) with mutations known to enhanced transmissibility or infectivity (such as the N501Y mutation in the S-protein), confirmed by epidemiological data (rapid spread in human populations), are of particular concern. Although all VOC investigated in this study have the N501Y mutation in common, it seems that they have emerged separately. For example, COH-VOC has the N501Y mutation similar to other VOC, and fell in clade GH with SA-VOC, but lacks most of the other S-protein mutations detected in SA-VOC, implying an independent evolutionary pathway. Along with the N501Y mutation in UK-VOC, it is speculated that the combination of the P681H mutation in the furin cleavage site and deletion of two amino acids at positions 69–70, which can alter the homology of the S-protein, is likely enhancing the transmissibility of SARS-CoV-2 and resulting in increased patient numbers [32,33]. Moreover, the SA-VOC with rapid spread contains the E484K mutation, in addition to N501Y, which can be also found in BR-VOC (highly mutated variant).

Among all possible vaccine candidates (such as attenuated and inactivated, subunit recombinant, viral vectors, and virus-like particles, VLP), the most promising candidates are mRNA based vaccines that were deployed by BioNTech/Pfizer and Moderna. Pfizer vaccine encodes RBD of SARS-CoV-2, while Moderna vaccine uses the full length of S-protein as an immunogen [18,34]. Due to the appetence of new mutant variants of SARS-CoV-2, a growing concern is the impact of viral genome changes, since some mutations alter both fitness and neutralization susceptibility of SARS-CoV-2 [35]. Therefore, our main focus in this study was on mutations in the S-protein, which plays a critical role in virus attachment to host cells and is the major viral antigen in the current vaccines. Thus, any changes in the S-protein structure or binding affinity could potentially compromise therapeutic or vaccine effectiveness through evading vaccine-induced immunity [36]. The four VOC analyzed in our study showed highest mutation rates in the ORF1a,b and S-protein, while no convergence were observed in M, ORF6, ORF7b, and ORF10.

In silico docking analysis on RBD mutations found in novel VOC aid in the understanding whether or not mutations in RBD strengthen the binding affinity and amino acid interactions in RBD-hACE2 complex, leading to more infectious variants. Therefore, we employed MM/GBSA as an efficient and reliable method to predict the FEB of the RBD-hACE2 docked complexes [37,38]. Accordingly, a large negative electrostatic FEB is in favor of binding and complex formation, however, a positive polar solvation FEB dampens the binding affinity and complex formation [39].

Any mutations at position N501 (in the key residue of RBD) and E484 (in interface recognition loop) are important, since mutations in these sites may change the binding affinity between RBD and hACE2. In silico analysis demonstrated that the Y501 mutation in all VOC forms shorter H-bonds (length ranging from 2.82 to 2.94 Å) than its counterpart in WT variant residue N501, thus, establishing a stable interaction between RBD and hACE2. Further, the Y501 mutation in VOC has more negative contribution to the total FEB (−7.18 ± 0.17 kcal/mol) than its counterpart in WT variant residue N501 (−2.92 kcal/mol). Therefore, combining FEB and molecular interaction data, the N501Y mutation in RBD strengthens binding affinity of SARS-CoV-2 RBD to hACE2. Our data is consistent with previous studies describing the possibility of strengthen infectivity following the of N501Y mutation [31].

The residue E484 in WT, UK-VOC, and COH-VOC makes a weak H-bond (length of 3.17–3.19 Å) with K31 on hACE2. The residue K484 in SA-VOC does not appear to interact with hACE2, whereas its counterpart in BR-VOC makes a strong H-bond (length of 2.60 Å) with E75 on hACE2, thus, forming a salt-bridge. Furthermore, the residue E484 in WT, UK-VOC, and COH-VOC contributed positively to the total FEB by +1.34 kcal/mol (unfavorable contribution) and the corresponding residue in mutant variants, which is residue K484 lowered this positive contribution to -0.59 kcal/mol for SA-VOC and −2.17 kcal/mol for BR-VOC. Therefore, in light of FEB and molecular interaction results, it seems that the E484K mutation in SA-VOC has no significant effect on binding affinity between SARS-CoV-2 RBD and hACE2. Nevertheless, there are evidences that the E484K mutations may give the virus new features to evade antibodies that neutralize the SARS-CoV-2 [10,40]. Conversely, the E484K mutation in BR-VOC potentially strengthens binding affinity of SARS-CoV-2 RBD to hACE2.

The K417 residue in WT, UK-VOC and COH-VOC contributes significantly to the total FEB (−2.76 ± 0.01 kcal/mol), while forming a strong H-bond (length of 2.59 Å) with residue D30 on hACE2 and forming a salt-bridge. Therefore, this salt-bridge is found to be important for the stability of the RBD-hACE2 complex in WT, UK-VOC and COH-VOC, which is consistent with previous findings [39]. This is worth noting that the residue K417 has two different mutations, namely, N417 (+0.21 kcal/mol) and T417 (+0.22 kcal/mol) in SA-VOC and BR-VOC respectively, which has an unfavorable contribution in binding affinity. Further, due to the long distance between N/T417 and hACE2, molecular interface is not significant. As a result, the salt-bridge between position 417 on WT RBD and D30 is lost in SA-VOC and BR-VOC, since an electrostatic attraction can form when the distance between two oppositely charged amino acids is 4 Å or less apart [16]. Therefore, combining data from FEB and molecular interface, this might be assumed that the K417N/T mutations disfavor complex formation between RBD and hACE2, which is consistent with a previous study on SA-VOC [41]. Nevertheless, mutations at this residue seem to have a moderate impact on RBD-hACE2 binding affinity, as it is located in a region with a lower probability of contact, which is in line with a previous study describing the lower probability of contact in the K417N mutation [42]. Even though, the mutation K417T in BR-VOC would indirectly favor complex formation in BR-VOC by changing the conformation of RBM at the flexible loop, where K484 in BR-VOC forms a strong H-bond with E75.

We predicted the binding affinity of the RBD-hACE2 in a recently emerged variant in California, known as CAL-20C (lineage B.1.427 and B.1.429). The CAL-20C with L452R mutation in RBD is thought to be a more transmissible variant mainly due to epidemiological data (37% of all samples collected in California in January 2021 was CAL-20C) [43]. The calculation of FEB showed that the residue L452 in WT variant and R452 in CAL-20C contributes to the total binding energy by +0.01 kcal/mol and −0.96 kcal/mol respectively. As it can be seen, neither L452 nor R452 have a significant contribution to the total binding energy, however, L452R mutation is slightly in favor of complex formation in CAL-20C by lowering the binding energy (Supplementary Figure S1). We have not considered this variant as a VOC, since the increased number of infected cases with CAL-20C is not associated with any significant mutation signatures (e.g., N501Y and E484K) like what can be seen in other VOC. Thus, it makes sense to consider novel variants of SARS-CoV-2 as VOC if mutations in RBM significantly strengthen binding affinity (e.g., N501Y) in RBD-hACE2 complex (genetic data), while associated with rapid spread in human populations (epidemiological data).

Until now, developed vaccines for SARS-CoV-2 seem to be effective against novel variants, as induced immunity by vaccines are based on polyclonal antibody production through targeting several parts of the S-protein [44]. However, once vaccination has started, there will be selective pressure that would be in favor of the emergence of novel variants capable of escaping the immune system through selecting for “escape mutants’. Therefore, genomic surveillance should be fostered to find and monitor new emerging SARS-CoV-2 variants before they become a public health concern.

Further, it is very important in future to determine the effect of mutations in VOC on the strength and specificity of neutralizing antibodies in humans [45]. To this, it will also be important to consider the original antigenic sin whereby CD4 T cell immunity generated by vaccination against the WT spike protein may be prejudiced to WT N501 memory responses favoring immune escape.

## 5. Conclusions

The genomic epidemiology of SARS-CoV-2 will help to distinguish variants of significant concern from other variants with less public health significant. This study uncovered that mutations in VOC have a favorable contribution to the total binding energy, particularly due to N501Y and E484K mutations, underlying the higher affinity of novel VOC for hACE2 compared to the WT isolate. The higher affinity of novel SARS-CoV-2 VOC for binding with hACE2 correlates with higher human-to-human transmissibility of VOC compared to the WT isolate. Therefore, to understand the impact of mutations on viral fitness and its genome modification in response to vaccination, it is necessary to continue to survey the evolution of SARS-CoV-2.

## Figures and Tables

**Table 1:** Nonsynonymous mutations of four recent VOC (UK-VOC, SA-VOC, COH-VOC, B.J-VOC) are compared with SARS-CoV-2 reference sequence as WT variant. RBD and O-linked glycan domain (OGD) are colored as blue and yellow respectively. Mutations are colored as red.

Gene		Sequences	Ref. Seq.	UK-VOC	SA-VOC	COH-VOC	BR-VOC
	aa Position						
ORF1ab	NSP2-4		R	R	C	R	R
	NSP2-85		T	T	T	I	T
	NSP3-181		T	T	T	I	T
	NSP3-183		T	I	T	T	T
	NSP3-256		A	A	A	V	A
	NSP3-370		S	S	S	S	L
	NSP3-837		K	K	N	K	K
	NSP3-890		A	D	A	A	A
	NSP3-977		K	K	K	K	Q
	NSP3-1412		I	T	I	I	I
	NSP5-89		L	L	L	F	L
	NSP5-108		P	P	P	S	P
	NSP6-106-108		SGF	DEL	SGF	SGF	DEL
	NSP6-258		G	G	G	E	G
	NSP12-323		P	L	L	L	L
	NSP13-168		E	E	D	E	E
	NSP13-341		E	E	E	E	D
	NSP14-28		S	S	C	S	S
	NSP14-129		N	N	N	D	N
	NSP15-205		P	P	L	P	P
	NSP16-216		R	R	R	C	R
S	S-18		L	L	L	L	F
	S-20		T	T	T	T	N
	S-26		P	P	P	P	S
	S-69-70		HV	DEL	HV	HV	HV
	S-80		D	D	A	D	D
	S-138		D	D	D	D	Y
	S-144		Y	DEL	Y		Y
	S-190		R	R	R	R	S
	S-215		D	D	G	D	D
	S-241-243		LLA	LLA	DEL	LLA	LLA
S-RBD	S-417		K	K	N	K	T
	S-484		E	E	K	E	K
	S-501		N	Y	Y	Y	Y
	S-570		A	D	A	A	A
	S-614		D	G	G	G	G
	S-655		H	H	H	H	Y
S-OGD	S-681		P	H	P	P	P
	S-701		A	A	V	A	A
	S-716		T	I	T	T	T
	S-982		S	A	S	S	S
	S-1027		T	T	T	T	I
	S-1118		D	H	D	D	D
	S-1176		V	V	V	V	F
ORF3	NS3-57		Q	Q	H	H	Q
	NS3-171		S	S	L	S	S
	NS3-172		G	G	G	V	G
	NS3-253		S	S	S	S	P
E	E-71		P	P	L	P	P
M	-		-	-	-	-	-
ORF6	-		-	-	-	-	-
ORF7a	NS7a-120		T	T	T	I	T
ORF7b	-		-	-	-	-	-
ORF8	NS8-24		S	S	S	L	S
	NS8-52		R	I	R	R	R
	NS8-73		Y	C	Y	Y	Y
	NS8-92		E	E	E	E	K
N	N-3		D	L	D	D	D
	N-67		P	P	P	S	R
	N-80		P	P	P	P	R
	N-199		P	P	P	L	P
	N-203		R	K	R	R	K
	N-204		G	R	G	G	R
	N-205		T	T	I	T	T
	N-235		S	F	S	S	S
ORF10	-		-	-	-	-	-
Total number	Mutation/deletion		NA	20	18	17	23

## Supplementary Materials

The following are available online at https://www.mdpi.com/article/10.3390/microorganisms9050926/s1, Figure S1: Structure of the SARS-CoV-2 RBD (green) docked with hACE2 (blue).
